# Supplementary material for: The discovery of novel noncoding RNAs in 50 bacterial genomes
Source: Nucleic Acids Res. 2024 Apr 22;52(9):5152–65. doi: 10.1093/nar/gkae248 (PMC11109978; doi:10.1093/nar/gkae248)
Supplement: gkae248_Supplemental_Files [file gkae248_supplemental_files.zip › Narunsky 50 Genomes RNA Motifs Files.zip!/Publication/54-P_aestuarii/Figures/LRC-54-11-NC_011059_1_IGR_37_1239626_1239834.sto.pdf]

LRC-54-11-NC_011059_1_IGR_37_1239626_1239834.sto.r2r

5′-A C U U G A U A A G G A G G C C U G A C G C G C C U C A A U G C U U C U C A A G A G G U G A G C C C G G A U G U U C A ● G G U U A A A C A G C A U U G A U C A G C A C G U C ● C G C C A G A C U C A A C C G A G A U G C G G A A A A U C ● A C C A U G Y U G U C A U G A U G G C G G C A U U G G U U G C C G A A U A A U C U C A U A ● G C A A U A G G A U G U G A A A A A G C A G C C C U C C C G A U G C A U G A C A G G C A A
